# The effects of an acute Tai Chi on emotional memory and prefrontal cortex activation: a fNIRS study

**DOI:** 10.3389/fnbeh.2024.1520508

**Published:** 2025-01-22

**Authors:** Haining Wang, Yujiang Guo, Hao Fan, Zhihao Chen, Shumeng Liu, Longfei Zhao, Yonggang Shi

**Affiliations:** ^1^Department of Martial Arts and Traditional Ethnic Sports, Henan Sport University, Zhengzhou, Henan, China; ^2^School of Physical Education, Zhengzhou University, Zhengzhou, Henan, China; ^3^Faculty of Psychology, Beijing Normal University, Beijing, China; ^4^Faculty of Physical Culture, Gdansk University of Physical Education and Sport, Gdansk, Poland

**Keywords:** Tai Chi, emotional memory, near-infrared spectroscopy, acute exercise, prefrontal cortex

## Abstract

**Objective:**

Acute exercise has the potential to influence emotional memory and cortical hemodynamics, but the specific effects depend on the type of exercise. This study aimed to determine whether acute Tai Chi practice enhances emotional memory and prefrontal cortex activation compared to cycling and a control condition.

**Methods:**

Using a within-subjects crossover design, 36 healthy university students completed three interventions: Tai Chi, cycling, and a resting control condition. Emotional memory performance was assessed before and after each intervention, and cortical hemodynamics were measured using functional near-infrared spectroscopy (fNIRS). The correlation between oxyhemoglobin (Oxy-Hb) concentration in the prefrontal cortex and emotional memory accuracy was analyzed.

**Results:**

Compared to cycling and the control group, the Tai Chi intervention showed: (1) a significantly higher accuracy of positive emotional memory; (2) a greater increase in Oxy-Hb concentration in the left dorsolateral prefrontal cortex (L-DLPFC) during positive emotional memory tasks; (3) a stronger positive correlation between Oxy-Hb concentration in the L-DLPFC and emotional memory accuracy. In contrast, cycling improved positive emotional memory accuracy to a lesser extent, while the control group showed no significant changes.

**Conclusion:**

Tai Chi, compared to cycling and rest, significantly enhanced positive emotional memory and L-DLPFC activation. These findings highlight the unique potential of Tai Chi to improve emotional memory through increased cortical activation, suggesting its effectiveness as a cognitive-emotional intervention.

## 1 Introduction

Emotional memory, encompassing both negative and positive emotional experiences, involves the intricate processes of encoding, storing, retrieving, and extracting emotional information (Ledoux, 1993). Emotionally charged events or information are often more vivid and easier to recall than neutral ones, a phenomenon known as emotional memory enhancement (EME). Emotional stimuli, whether positive or negative, capture more attention and evoke higher arousal, facilitating deeper encoding and stronger memory consolidation (Zhou et al., 2023). Emotional memory is closely intertwined with an individual’s psychological wellbeing. Studies have shown that stress-related mental disorders (Vos et al., 2016), such as depression and anxiety, can lead to heightened recollection of negative information (negative emotional memory), resulting in memory biases (Beck, 1979; Disner et al., 2011). Therefore, it is crucial to implement interventions (e.g., exercise or meditation) that reduce negative memories while enhancing positive ones in order to restore balance in emotional memory.

Research has revealed that exercise plays a crucial role in regulating cognition, including the enhancement of learning and memory processes (Herold et al., 2018; Zheng et al., 2023). Evidence further suggests that the effects of exercise may interact with the emotional content of encoded material (Jentsch and Wolf, 2020), consistent with growing evidence that exercise can enhance emotional memory (Loprinzi et al., 2019). In the context of short-term, single-session exercise interventions, acute exercise has been shown to enhance memory tasks (Skriver et al., 2014) and facilitate better recall of emotional images (Weinberg et al., 2014). Recent studies also confirm the role of acute exercise in modulating neural mechanisms that enhance emotional memory function (Loprinzi et al., 2022; Loprinzi et al., 2019). Additionally, studies have found that the enhancement of emotional memory relies on the interaction between the amygdala and hippocampus during the memory consolidation process, with exercise shown to positively influence this interaction, thereby enhancing emotional memory. This highlights the complex neural pathways involved in both emotional memory encoding and retrieval, underscoring the combined roles of the prefrontal cortex, amygdala, and hippocampus in emotional memory processing (Lin et al., 2015; Zhang et al., 2024).

Alterations in cerebral blood flow, particularly within the prefrontal cortex (PFC), have been identified as potential mechanisms underlying the relationship between acute exercise and emotional memory (Herold et al., 2018). The activation of the PFC is closely related to behavioral control in tasks involving exercise, memory, and emotion regulation (Basso et al., 2015; Silvers et al., 2015; Venkatraman and Huettel, 2012). When individuals encounter tasks that encompass cognitive and socio-emotional domains, particularly in guiding memory during the encoding of emotional experiences, the PFC plays a crucial role in constructing event meaning and regulating emotional intensity (Kensinger and Ford, 2021; Murty et al., 2010). Therefore, during the successful encoding of emotional memory, PFC regions involved in emotional processing, memory formation, attention, and perceptual processing are consistently activated. A study using transcranial magnetic stimulation (TMS) demonstrated that changes in PFC activity mediate factors contributing to successful encoding (Kensinger et al., 2003), with consistent activation observed in the dorsolateral prefrontal cortex (DLPFC) during the retrieval of emotional memory (Dahlgren et al., 2020). The L-DLPFC specifically plays a key role in regulating the intensity of emotional responses, such as those related to fear or happiness, and contributes to the regulation of emotional memory through its interactions with the amygdala. This region helps modulate emotional reactivity by filtering out irrelevant emotional stimuli and enhancing the processing of positive emotional content (van Kleef et al., 2022). Studies also suggest that the L-DLPFC is involved in the cognitive appraisal of emotional experiences, influencing how these emotions are encoded into memory (Dolcos et al., 2017). The DLPFC is closely associated with “cognitive” or “executive” functions, such as working memory, intention formation, goal-directed action, maintenance and operation of attentional control (Miller and Cohen, 2001), as well as the processing of emotional information (Langenecker et al., 2005). The DLPFC is essential for modulating threat-related expressions during the process of memory reconsolidation (Battaglia et al., 2024; Borgomaneri et al., 2020). Research has shown that this region contributes to emotional regulation by influencing amygdala activity, thereby dampening responses to perceived threats (Amaral, 2002; Delgado et al., 2008). Moreover, repetitive transcranial magnetic stimulation (rTMS) studies have highlighted the role of the L-DLPFC in enhancing the recognition of emotionally positive and highly arousing words (Balconi and Cobelli, 2015). These findings indicate that prefrontal activation plays a significant role in the processing and recognition of emotional memories. The L-DLPFC exhibited greater activation during the evaluation of positive images compared to negative images, as revealed by an fMRI study (Dolcos et al., 2004; Kensinger and Schacter, 2006). Furthermore, extensive neuroimaging studies have investigated the encoding and retrieval of emotional episodic memories, providing valuable insights into the neural basis of emotional memory (Dolcos et al., 2017). In terms of the neural correlates of cognitive tasks before and after acute exercise, several studies have reported that acute exercise enhances executive function and increases prefrontal cortex (PFC) activation in both young and older adults (Banich et al., 2000; Hyodo et al., 2012; Tsujii et al., 2013; Yanagisawa et al., 2010). Given the L-DLPFC’s role in both cognitive control and emotional regulation, its enhanced activation following acute exercise may explain the improvements in emotional memory observed in this study. Additionally, it is worth noting that the PFC, particularly the DLPFC, plays an important regulatory role in various cognitive processes involved in motor learning (Chambon et al., 2011). Given that emotional memory is also a cognitive function (LaBar and Cabeza, 2006), there seems to be a connection between acute exercise and emotional memory, with the PFC significantly involved in both processes.

Tai Chi Chuan (TCC), originating from Chinese Taoist philosophy, has been practiced for centuries (Osypiuk et al., 2018). TCC integrates cognitive training and mindfulness meditation, both of which are positively linked to physical and psychological wellbeing (Vestal, 2017). By combining gentle, deliberate movements with diaphragmatic breathing, TCC promotes mental tranquility, enhances posture and respiration, improves bodily awareness, and sustains mental focus (Miller and Taylor-Piliae, 2014). Beyond its general benefits, TCC uniquely merges mindfulness and movement, which synergistically influence cognitive processes such as attention and memory—key components of emotional memory. Mindfulness-based practices have been shown to increase positive emotions and encourage continued engagement in such exercises (Garland et al., 2017). Theoretically, mindfulness-based practices enhance the positive valence system associated with approach-oriented behaviors by improving emotional awareness, regulating emotional reactivity, increasing the use of cognitive reappraisal, and modifying reward processing (Wielgosz et al., 2019). These benefits are linked to specific physiological activity patterns and regional brain activation, particularly in areas such as the dorsolateral prefrontal cortex (DLPFC) and orbitofrontal cortex (OFC), which play key roles in supporting positive emotional states (Weng et al., 2013). Similarly, growing evidence underscores TCC’s significant role in fostering positive emotions, reducing negative emotional states such as depression, anxiety, and mental stress, and enhancing working memory (Kong et al., 2019; Wang et al., 2023; Wang et al., 2023; Xie et al., 2021). Neuroimaging studies provide further support for TCC’s cognitive benefits. For instance, resting-state functional magnetic resonance imaging (R-fMRI) studies using the regional homogeneity (ReHo) method have revealed structural differences in key brain areas, such as the prefrontal cortex (PFC), between TCC practitioners and non-practitioners. These findings indicate that TCC may induce cortical thickness changes comparable to those associated with aerobic exercise and meditation (Wei et al., 2013; Xie et al., 2019; Yao et al., 2021). Furthermore, Near-infrared spectroscopy (fNIRS) studies offer additional insights, showing that TCC practice enhances HbO2 concentration in the PFC and increases functional connectivity between the left and right PFC during sessions (Qi et al., 2023). A 10-month longitudinal study revealed that TCC training reduced negative emotions, which was associated with enhanced functional connectivity between the prefrontal cortex and key emotion-related regions, as well as increased gray matter in the prefrontal cortex (Wei et al., 2016). These findings emphasize TCC’s potential to facilitate attentional control and emotional memory consolidation by engaging neural mechanisms associated with mindfulness and movement.

Although several studies have reported positive effects of acute exercise on emotional memory function (Jentsch and Wolf, 2020; Keyan and Bryant, 2017b, 2017a; Wade and Loprinzi, 2018), inconsistent findings and a lack of systematic comparison between its effects on positive and negative emotional memories are evident in current research. Additionally, variations in experimental design, participant characteristics, exercise type, and intensity across different studies hinder direct comparison and synthesis of results. Current understanding of the mechanisms underlying the acute exercise-induced enhancement of cognitive performance is incomplete (Pontifex et al., 2019). Given the existing evidence suggesting that moderate-intensity exercise can enhance emotional memory, previous research has predominantly focused on exercise modalities such as brisk walking, jogging, and power cycling, resulting in relatively uniform intervention approaches. This type of research could provide valuable insights into the potential applications of acute exercise interventions for diverse populations, including older adults and individuals with clinical conditions. Furthermore, fNIRS, a non-invasive and portable neuroimaging technique, offers a promising approach to studying the neural mechanisms underlying acute exercise effects. By detecting oxygenated (HbO_2_) and deoxygenated (HbR) hemoglobin, fNIRS measures changes in cortical hemodynamics with high temporal resolution, allowing precise tracking of brain activity during tasks. Compared to traditional neuroimaging methods like functional magnetic resonance imaging (fMRI), fNIRS is more cost-effective, tolerant of movement artifacts, and suitable for naturalistic environments (Herold et al., 2018). These features make it particularly advantageous for studies involving physical activity such as Tai Chi Chuan (TCC), enabling researchers to investigate how changes in prefrontal cortex (PFC) oxygenation mediate improvements in emotional memory. This study aims to investigate which type of acute exercise is most effective in improving emotional memory among individuals. It hypothesizes that the integration of mindfulness and movement in TCC will result in improved recall of positive emotional content and greater prefrontal HbO_2_ activation, highlighting its unique cognitive and emotional benefits. By combining mindfulness with physical exercise, TCC offers a distinctive mind–body approach to enhancing cognitive functions, including emotion regulation, attentional focus, and memory performance.

## 2 Materials and methods

### 2.1 Participants

Using G*Power 3.1 software, the required sample size was calculated to be 33 participants (*α* = 0.05, power = 0.85, effect size = 0.25) based on prior studies investigating the effects of acute exercise on cognitive and emotional outcomes (Faul et al., 2007; Nakamura et al., 2023). The study design involved three groups with four levels of factors, which were considered when determining the sample size to ensure sufficient statistical power. Participants were recruited from a university and consisted of non-sports major college students aged 18–25, all right-handed, with normal or corrected-to-normal vision and stable daily routines. They had no history of psychiatric disorders or medication use. To minimize expectation effects, specific hypotheses were not disclosed during recruitment. Informed consent was obtained from each participant, who signed consent forms. To account for potential attrition, the sample size was increased by 20%, resulting in the recruitment of 42 students. Eligibility was assessed using the Physical Activity Readiness Questionnaire (PAR-Q) to ensure low risk for adverse events during physical activity. During testing, two participants could not attend due to illness, and two others failed to understand the rules of the emotional memory test, preventing completion. Ultimately, 38 students completed all procedures. After processing the near-infrared spectroscopy (NIRS) data, two participants were excluded due to poor data quality caused by excessive artifacts. Thus, the final analysis included data from 36 participants (16 males and 20 females). Written informed consent was obtained from all participants, and ethical approval was granted by the Institutional Review Board of Henan University of Sport, adhering to the principles outlined in the Declaration of Helsinki.

### 2.2 Questionnaire measures

Demographic, behavioral, and psychological variables were assessed at baseline. Demographic variables included age and BMI, with the latter used to classify obesity and underweight status. Considering that emotional memory information might affect responses to emotional stressors and that related pathologies might impact emotional experiences, these features were also measured at baseline. Prior to group assignment, participants completed the Positive and Negative Affect Schedule (PANAS) as well as the Short Form of the Trait Mindfulness Questionnaire (Osman et al., 2016; Watson et al., 1988). All scales and questionnaires used in this study were administered in Chinese language and demonstrated robust psychometric properties within Chinese samples (Song et al., 2023; Wu et al., 2024).

Assessors were unaware of the participants’ group assignments, and grouping and ordering were conducted using a random number table generated in Excel under balanced sex conditions to minimize bias in the assessment of outcomes. Baseline demographic characteristics (e.g., age, behavioral traits, and psychological traits) were comparable across all three groups (Table 1). All experiments were conducted between 2:00 PM and 5:00 PM. Participants arrived at the laboratory after lunch and underwent a brief period of rest. Subsequently, each participant’s resting heart rate was measured using a Polar heart rate monitor. Prior to and following either a 30-min exercise or a 30-min rest control condition, participants completed an emotional memory task while undergoing two sessions of NIRS recordings. For heart rate recovery assessment, the second task was repeated 10 min after completion of the exercise/control condition (Byun et al., 2014). To minimize motion artifacts during NIRS measurements, all participants received instructions to keep their head position as still as possible.

**Table 1:** Participant demographics (Mean ± SD).

Group	Control group	Aerobic cycling intervention group	Tai Chi intervention group	*F*
Age (years)	21.583 ± 0.900	21.167 ± 0.577	22.000 ± 1.279	0.570
Sex (male/female)	5/7	5/7	6/6	
Height (cm)	171.546 ± 7.647	170.833 ± 10.179	174.250 ± 6.943	0.550
Weight (kg)	69.2 ± 18.534	62.75 ± 10.575	67.833 ± 13.093	0.656
BMI (kg/m^2^)	21.685 ± 3.946	21.364 ± 1.696	22.207 ± 3.297	0.222
State mindfulness	15.583 ± 5.756	19.167 ± 4.549	16.667 ± 3.892	1.761
Positive emotion state	31.750 ± 6.797	33.667 ± 4.418	32.833 ± 4.988	0.367
Negative emotion state	21.500 ± 6.417	24.917 ± 8.857	18.417 ± 7.537	2.157

### 2.3 Emotional memory test

This study assesses the emotional memory of college students using a block design and the established “study-recognition” paradigm, with moderate adjustments for NIRS research methodologies (Basden et al., 1993; Loprinzi et al., 2022). In the encoding phase, participants view 120 pictures (60 negative and 60 positive), each followed by a prompt to either “remember” or “forget.” During “remember” prompts, participants are instructed to recall the picture, while during “forget” prompts, they aim to forget it. After this phase, participants engage in a distraction task for 3 min, performing calculations (addition, subtraction, multiplication, and division) using a pre-listed sheet. They are encouraged to complete as many calculations as possible before time expires. Following the distraction task, an additional set of 120 new pictures (60 negative and 60 positive) is introduced as interference. The total pool of 240 pictures is evenly divided for testing, with an intermission provided between groups. All stimuli consist of images sourced from the International Affective Picture System (IAPS) and are presented in a pseudo-randomized sequence (Lang et al., 1999).

All memory task demonstrations and behavioral data collection were conducted using E-Prime 3.0 software (Psychology Software Tools Inc., Sharpsburg, PA, USA). During the encoding phase, the emotional memory test consists of two conditions: positive and negative emotion memory. Each trial begins with a fixation point displayed for 1,000 milliseconds, followed by a “remember” or “forget” instruction for 2000 milliseconds. A stimulus image is then shown for 2000 milliseconds, followed by a fixation point for another 1,000 milliseconds. Five images are presented in each block, followed by a 15-s rest period to collect hemodynamic data from the regions of interest (ROIs). Immediately after the learning phase, participants complete a 3-min distraction task involving calculations (addition, subtraction, multiplication, and division) on pre-listed sheets, aiming to perform as many calculations as possible before time expires. In the testing phase, participants judge whether each image was previously seen. They press the “K” key for new images and the “S” key for old images. After understanding the procedure, they start the test by pressing the “space” key. A fixation point appears for 1,000 milliseconds, followed by randomly presented images requiring responses. Each image is displayed for a maximum of 4,000 milliseconds. The testing phase includes 240 images: 120 from the learning phase and 120 new interference images (60 positive and 60 negative), evenly divided into two groups with a rest period in between. Behavioral data (reaction time and accuracy) and hemodynamic data are collected during the test session. Prior to the experiment, participants receive instructions and training, which includes a practice session of two trials covering both encoding and testing phases involving positive and negative emotions. Following this practice session, there is a 15-s rest period before the formal test begins.

During the experiment, the emotional memory test and behavioral data collection were conducted on a Dell desktop computer with a 3.0 GHz CPU running Windows 10. The 23.8-inch display featured a high resolution of 1920 × 1,080 for optimal clarity and comfort. All protocols were controlled using E-Prime 3.0 software to ensure precise task execution. Images had resolutions of either 433 × 315 or 315 × 433 pixels. To minimize discomfort from negative images, only those with valence scores between 2 and 3.5 and arousal scores between 5 and 7 were selected. The screen background during testing was uniformly black, and participants were instructed to maintain a distance of approximately 60 cm from the monitor.

### 2.4 fNIRS data collection

**Figure 1:**
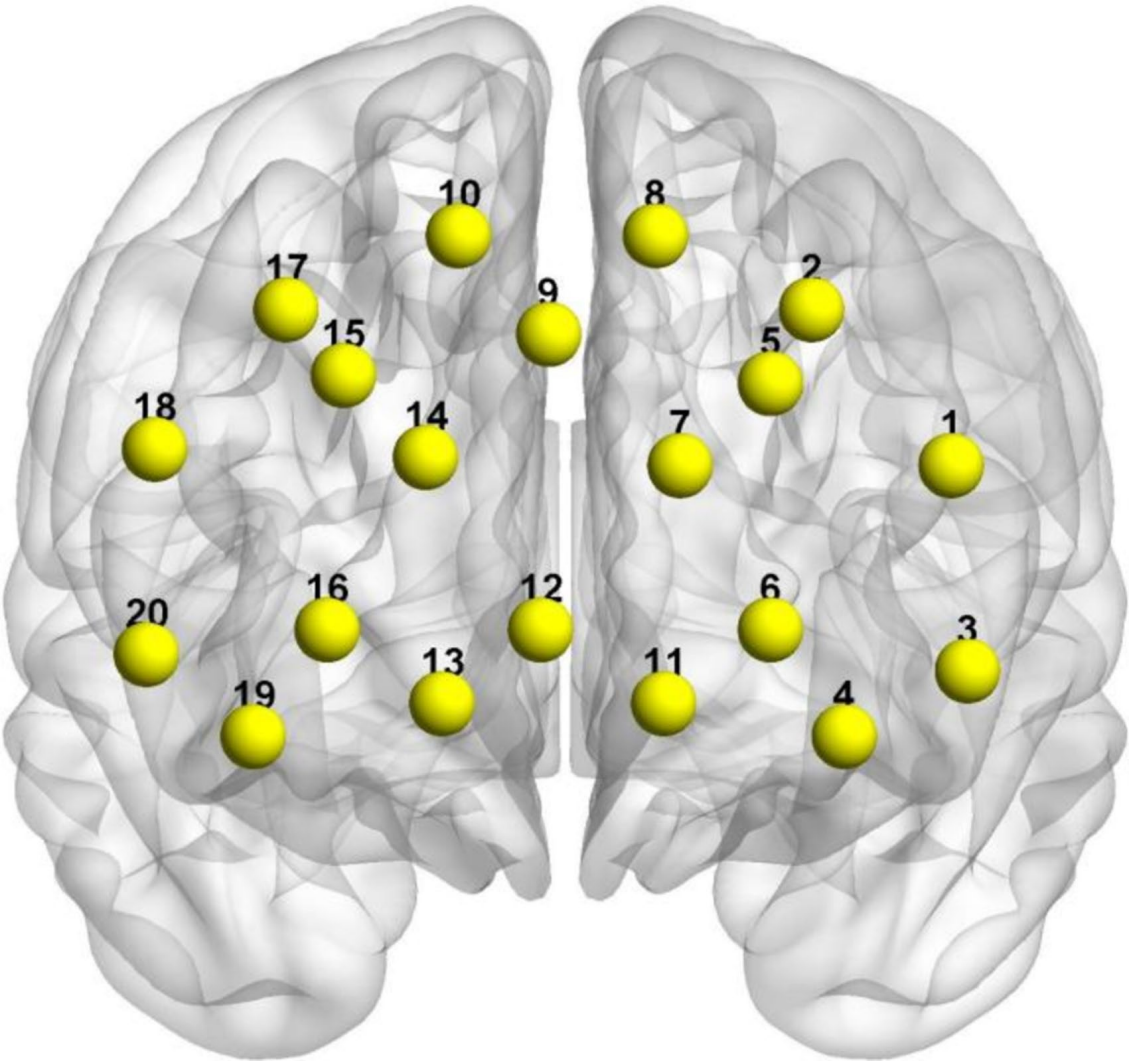
MNI spatial coordinates and channel distribution on the cerebral cortex.

In this experiment, we utilized the NIRScout desktop fNIRS system, manufactured by NIRX in the United States, which is specifically designed for capturing localized hemodynamic signals in the brain during both resting state and task execution. To ensure precise probe placement, we adhered to the internationally recognized 10–20 system and conducted meticulous calibration using specialized instruments and corresponding templates to accurately covered the PFC, a critical region of interest in the brain. The sampling frequency of the equipment was set at 10 Hz to capture changes in cortical hemodynamics. Simultaneously, a NIRSport2 NIRS system (NIRx Medical Technologies, LLC) was used to record cerebral hemodynamic activity with a primary focus on PFC activity. The NIRx system employed two wavelengths of avalanche photodiode source (760 and 850 nm) to obtain light intensity signals at a sampling frequency of 10.2 Hz. The modified Beer–Lambert law was applied to convert these light intensity signals into concentration changes of OxyHb and deoxyhemoglobin (DeoxyHb). This study utilized an 8 × 7 probe template referencing the 10–20 system for electrode placement with its position centered over the prefrontal area (Figure 1).

The three-dimensional coordinates for each channel location were obtained using a 3D head positioning modeling system (Patriot, Polhemus, USA). Spatial registration was performed using the NIRS_SPM package in Matlab. Based on the spatial distribution of the 20 channels, we defined 4 Regions of Interest (ROIs). The mapping of respective channels to brain regions is presented in Table 1. By referring to Tables 1, 2, as well as using Brodmann areas as a standard for division, we classified the channel locations into four primary regions: L-DLPFC, right dorsolateral prefrontal cortex (R-DLPFC), left frontopolar area, and right frontopolar area. These selected ROIs aimed at investigating activation patterns within these specific brain areas under different experimental conditions. Brain region mapping was performed using the BrainNet Viewer package in Matlab (Xia et al., 2013) (Table 3).

**Table 2:** MNI coordinates of fNIRS channels and corresponding Brodmann areas.

Channel	MNI coordinates			Brodmann areas	
	X	Y	Z	Brain regions	Probability
CH01	−47	30	40	Dorsolateral prefrontal cortex	0.626
CH02	−28	32	55	Dorsolateral prefrontal cortex	0.402
CH03	−53	34	21	Dorsolateral prefrontal cortex	0.869
CH04	−50	46	8	Dorsolateral prefrontal cortex	0.581
CH05	−29	42	45	Dorsolateral prefrontal cortex	0.982
CH06	−44	53	16	Dorsolateral prefrontal cortex	0.813
CH07	−24	61	29	Frontopolar area	0.772
CH08	−10	46	52	Dorsolateral prefrontal cortex	0.758
CH09	5	55	45	Dorsolateral prefrontal cortex	0.608
CH10	24	40	54	Dorsolateral prefrontal cortex	1.000
CH11	−28	67	0	Frontopolar area	1.000
CH12	0	68	9	Frontopolar area	1.000
CH13	15	73	−4	Frontopolar area	0.743
CH14	22	65	27	Frontopolar area	0.920
CH15	39	47	36	Dorsolateral prefrontal cortex	0.785
CH16	38	63	13	Frontopolar area	1.000
CH17	44	34	42	Dorsolateral prefrontal cortex	0.602
CH18	54	34	28	Dorsolateral prefrontal cortex	0.859
CH19	47	56	−3	Frontopolar area	0.730
CH20	56	40	5	Dorsolateral prefrontal cortex	0.418

**Table 3:** Channel ROI classification.

Regions of interest (ROIs)	Channels
Left dorsolateral prefrontal cortex, L-DLPFC	CH01, CH02, CH03, CH04, CH05, CH06, CH08
Right dorsolateral prefrontal cortex, R-DLPFC	CH09, CH10, CH15, CH17, CH18, CH20
Left Frontopolar area, RFPA	CH07, CH11, CH12
Right Frontopolar area, LFPA	CH13, CH14, CH16, CH19

### 2.5 Exercise intervention

**Figure 2:**
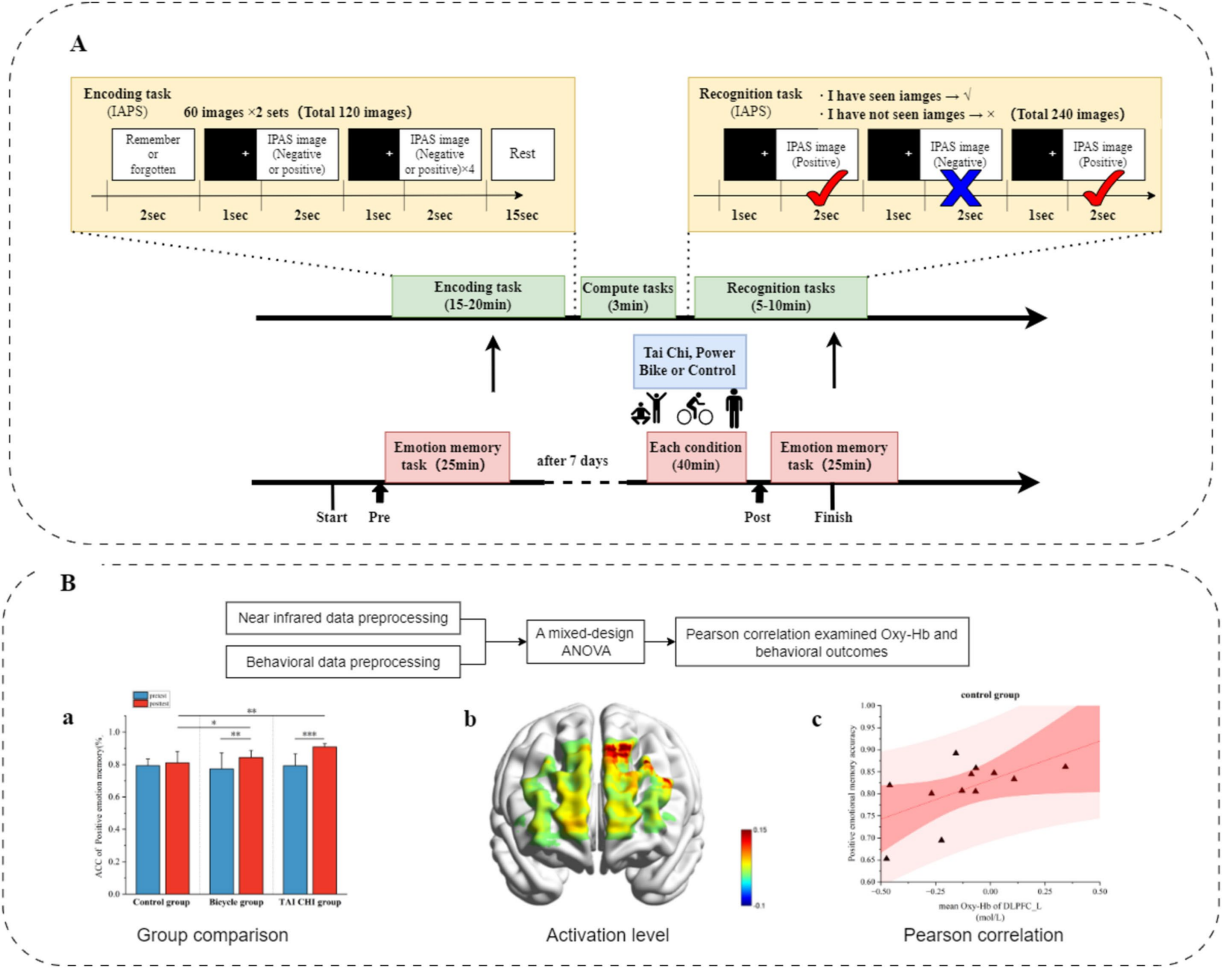
Overall experimental process. **(A)** Experimental paradigm and process arrangement. **(B)** Data processing.

In order to mitigate practice effects, the exercise intervention was implemented with a 1-day interval following the emotional memory test. *Acute Tai Chi (Eight Methods and Five Steps) Exercise intervention protocol:* the Eight Methods and Five Steps routine, developed by Professor Shaojun Lü based on Tai Chi principles that encompass philosophy, medical theory, and boxing theory as well as basic training rules such as relaxation, stillness, along with core elements including form, intention, and qi, was selected as the exercise intervention method (Niu et al., 2023). This routine has gained significant recognition and application in neuroscience research. *Exercise intensity:* participants initially underwent a comprehensive cardiopulmonary health assessment. Following the American College of Sports Medicine’s classification of aerobic exercise intensity in healthy adults, moderate-intensity aerobic exercise was defined as HR = (220 - age) × 60–69% (Cui et al., 2019; Shen et al., 2021). The exercise intensity was consistently maintained at a moderate level. *Duration:* The total duration of the exercise session is 50 min, which includes a 10-min warm-up period, a 10-min cool-down period, and a 30-min practice session of the Eight Methods and Five Steps routine. Each cycle of the Tai Chi routine lasts approximately 3 min and is repeated for about 7–10 times, resulting in a total duration of around 30 min. *Power cycling exercise intervention protocol:* participants engaged in aerobic exercise using a power cycle machine (Ergo select 100 k model). This group was included to better distinguish the specific effects of Tai Chi from other forms of aerobic exercise. *Duration:* The exercise session lasted for 50 min, comprising a 10-min warm-up phase, followed by a moderate-intensity cycling period lasting for 30 min. This was then concluded with a cooling down phase lasting another 10 min. During the aerobic exercise lasting for 30 min, participants utilized a stationary ergometer to cycle at a moderate intensity while maintaining their heart rate (HR) between 60 and 69%. *Monitoring:* heart rate was continuously monitored using a Polar watch. If any participant exhibited signs of distress (such as pale skin or lips) or abnormal physiological signals (e.g., irregular heart rate) during the acute aerobic exercise, the trial was immediately terminated. *Control group:* the control group did not undergo any exercise intervention. Instead, participants remained in a state of quiet rest, seated comfortably for 50 min before conducting the post-test. To ensure comparable mental engagement across groups and minimize potential confounding factors, participants in the control group were asked to read neutral books (e.g., general knowledge or nature-themed content without emotional or cognitive bias) during the rest period. Participants were instructed to focus solely on the reading task and to avoid engaging in any other distracting activities during this time. After completing their respective interventions, all participants, including those in the control group, rested for 15 min before initiating the formal tests with the fNIRS device (Figure 2).

### 2.6 Data processing and statistics

#### 2.6.1 Data preprocessing

*Behavioral data:* the behavioral data collected from the participants were processed, merged, and organized using the E-Merge3 function in E-Prime 3.0 software. The merged data were imported into Excel for further preprocessing (Chen et al., 2018). This involved eliminating reaction times that deviated more than ±3 standard deviations from the mean. *fNIRS Data processing*: (1) Data quality check: a manual quality check was conducted on the fNIRS data to identify and mark noisy channels as bad channels. Noisy channels were identified based on a signal-to-noise ratio threshold (SNR < 3) and the presence of signal discontinuities or spikes indicative of motion artifacts. Motion artifact thresholds were set using a standard deviation threshold (±5 SD) from the mean signal to detect transient signal deviations. Participants whose data exhibited excessive motion artifacts or failed to meet the minimum SNR threshold were excluded from the analysis (Pinti et al., 2020). (2) Data preprocessing: initial unstable signals during the first and last 15 s of both resting state and task periods were excluded for each participant. The preprocessing of fNIRS data was conducted using NirsLab software, involving several steps: (1) Data import: importing the fNIRS data into NirsLab software. (2) Probe configuration: importing the probe template used in this study. (3) Task marking: adjusting the study intervals corresponding to the behavioral tasks. (4) Data truncation: extracting valid segments of data for analysis. Baseline correction was performed using a 2-s data prior to each task block initiation. Each block was expected to take approximately 15 s to complete (6 blocks per condition). The data from second 1 to second 15 after stimulus onset were averaged together. (5) Discontinuity Removal: Converting raw light intensity data into OxyHb concentrations using modified Beer–Lambert law principles, followed by bandpass filtering to eliminate respiratory and cardiac components outside of the range > 0.1 Hz and < 0.01 Hz, respectively. (6) Further Analysis in SPM: Analyzing the obtained data in SPM to derive OxyHb values for each channel, ultimately exporting Beta values. Oxy-Hb demonstrates a higher signal-to-noise ratio and greater sensitivity toward changes in brain oxygenation (Lindenberger et al., 2009; Strangman et al., 2002). Therefore, Oxy-Hb was chosen as the indicator to analyze blood oxygen concentration in subsequent analyses. After organizing the Beta data for all channels under all experimental conditions for each participant, the data were imported into SPSS software for variance analysis.

#### 2.6.2 Statistical analysis

Statistical analyses were performed using SPSS 27.0 software (IBM Inc., Chicago, IL, United States). Pearson correlation analysis was conducted to examine the relationship between Oxy-Hb concentration during emotional memory encoding and behavioral outcomes. A mixed-design ANOVA was implemented with a 3 (Tai Chi group, power cycling group, and control group) × 2 (pre-intervention, post-intervention) × 2 (positive emotion and negative emotion) design. In this analysis, the participant group served as the between-subjects variable, while pre- and post-intervention and emotional valence (positive vs. negative) were treated as within-subjects variables. The mixed-design ANOVA was used to investigate the main effects and interaction effects across these factors, providing a comprehensive understanding of their combined impact on both behavioral outcomes (e.g., recall accuracy and reaction time) and Oxy-Hb concentration. The decision to use this model aligns with standard practices in experimental designs that examine multifactorial influences on dependent variables. For the behavioral data, accuracy and reaction time (RT) were analyzed using the mixed-design ANOVA. For the fNIRS data, a similar approach was adopted to evaluate changes in Oxy-Hb concentration. *Post-hoc* tests, effect size calculations (partial eta squared, *η*^2^), and paired comparisons were conducted when significant main effects or interactions were observed. The Bonferroni correction was applied to adjust the alpha level for multiple comparisons, ensuring robust statistical analysis.

## 3 Results

### 3.1 Behavioral results

**Figure 3:**
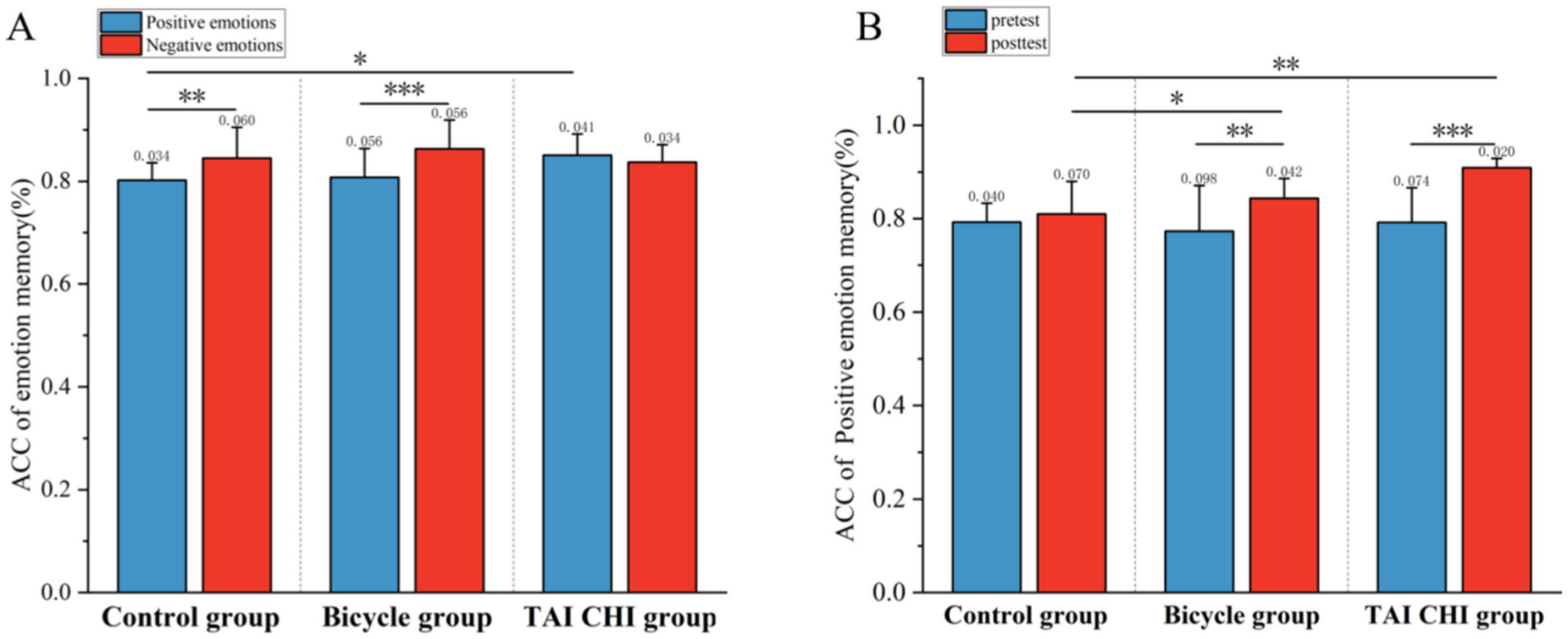
Behavioral results of emotional memory before and after testing for the Tai Chi group, cycling group, and control group. **(A)** Accuracy of positive emotional memory before and after testing; **(B)** Overall accuracy of positive and negative emotional memory. *p < 0.05; **p < 0.01; ***p < 0.001. Error bars represent SD.

The analysis of the accuracy of emotional memory, as depicted in Figure 3, revealed a significant main effect of time [*F*(1, 33) = 16.428, *p* < 0.001, *η*^2^ = 0.332], indicating a noteworthy improvement in memory accuracy following the intervention. Additionally, there was a significant main effect observed for emotional memory type [*F*(1, 33) = 13.807, *p* < 0.001, *η*^2^ = 0.295]. Furthermore, an interaction effect between time and group was found to be statistically significant [*F*(2, 33) = 4.599, *p* < 0.05, *η*^2^ = 0.218]. Subsequent simple effects analysis showed that both the Tai Chi group [*F*(1, 33) = 16.646, *p* < 0.001, *η*^2^ = 0.335] and the cycling group [*F*(1, 33) = 0.003, *p* > 0.05, *η*^2^ = 0.000] exhibited significant main effects; however, the control group did not demonstrate any notable effects [*F*(1, 33) = 16.646, *p* < 0.001, *η*^2^ = 0.335]. *Post-hoc* analysis indicated a significant increase in emotional memory accuracy after the intervention compared to before for both the Tai Chi group (0.890 ± 0.013 versus before: 0.796 ± 0.021, *p* < 0.001) and the cycling group (after: 0.870 ± 013 versus before: 0.801 ± 0.021, *p* < 0.01). The overall effect after the intervention was also significant [*F*(2, 33) = 7.713, *p* < 0.01, *η*^2^ = 0.319]. No significant difference in emotional memory accuracy was found between the cycling group (0.870 ± 0.013) and the Tai Chi group (0.890 ± 0.013, *p* > 0.05), but there were significant differences between the Tai Chi group and the control group (0.823 ± 0.013, *p* < 0.01), as well as between the cycling group and the control group (*p* < 0.05).

The interaction effect between emotional memory type and group was found to be significant [*F*(1, 33) = 8.574, *p* < 0.01, *η*^2^ = 0.342]. Simple effects analysis showed that the main effects were significant in the cycling group [*F*(1, 33) = 18.230, *p* < 0.001, *η*^2^ = 0.356] and the control group [*F*(1, 33) = 11.297, *p* < 0.01, *η*^2^ = 0.255], but not in the Tai Chi group [*F*(1, 33) = 1.428, *p* > 0.05, *η*^2^ = 0.041]. *Post-hoc* analysis revealed that the accuracy of negative emotional memory was higher than that of positive emotional memory in the cycling group (0.863 ± 0.015 versus 0.808 ± 0.013, *p* < 0.001) and in the control group (0.845 ± 0.015 versus 0.802 ± 0.013, *p* < 0.01). The main effect of positive emotional memory accuracy was found to be significant [*F*(2, 33) = 3.892, *p* < 0.05, *η*^2^ = 0.191]. Post-hoc analysis showed that the accuracy of positive emotional memory in the Tai Chi group (0.850 ± 0.013) was significantly higher than that in the control group (0.802 ± 0.013, *p* < 0.05) but not significantly different from that in the cycling group (0.808 ± 0.013, *p* > 0.05).

The analysis of reaction time for emotional memory revealed that the main effect of group was not statistically significant [*F*(2, 33) = 2.691, *p* > 0.05, *η*^2^ = 0.140]. Similarly, the main effect of time did not reach statistical significance [*F*(1, 33) = 0.437, *p* > 0.05, *η*^2^ = 0.013]. Furthermore, there was no significant interaction effect between time and group observed [*F*(2, 33) = 0.347, *p* > 0.05, *η*^2^ = 0.021]. Additionally, the main effect of emotional memory type was not significant [*F*(2, 33) = 2.491, *p* > 0.05, *η*^2^ = 0.070]. The interaction effect between emotional memory type and group was also non-significant [*F*(2, 33) = 0.098, *p* > 0.05, *η*^2^ = 0.052]. Similarly, the interaction effect between time and emotional memory type failed to reach statistical significance [*F*(1, 33) = 0, *p* > 0.05, *η*^2^ = 0]. Furthermore, the three-way interaction effect among time, group, and emotional memory type was not significant [*F*(2, 33) = 0.034, *p* > 0.05, *η*^2^ = 0.002] (Table 4).

**Table 4:** Descriptive statistical results of emotional memory accuracy and response before and after the experiment.

	Time	Variable	Control group (mean ± SD)	Bicycle group (mean ± SD)	Tai Chi group (mean ± SD)
			(*N* = 5 males/7 females)	(*N* = 5 males/7 females)	(*N* = 6 males/6 females)
Positive emotions	Pretest	Correctness rate (%)	0.793 ± 0.040	0.773 ± 0.098	0.792 ± 0.074
		Reaction time (ms)	952.994 ± 146.221	1093.660 ± 191.357	998.580 ± 141.919
	Posttest	Correctness rate (%)	0.810 ± 0.070	0.844 ± 0.042	0.909 ± 0.020
		Reaction time (ms)	1022.477 ± 130.162	1055.544 ± 144.624	1019.853 ± 85.200
Negative emotions	Pretest	Correctness rate (%)	0.854 ± 0.096	0.829 ± 0.098	0.800 ± 0.076
		Reaction time (ms)	907.62 ± 326.754	1055.544 ± 144.624	1011.327 ± 125.354
	Posttest	Correctness rate (%)	0.835 ± 0.076	0.896 ± 0.023	0.871 ± 0.037
		Reaction time (ms)	1007.353 ± 184.313	1041.518 ± 126.846	1030.127 ± 90.480

### 3.2 fNIRS results

**Figure 4:**
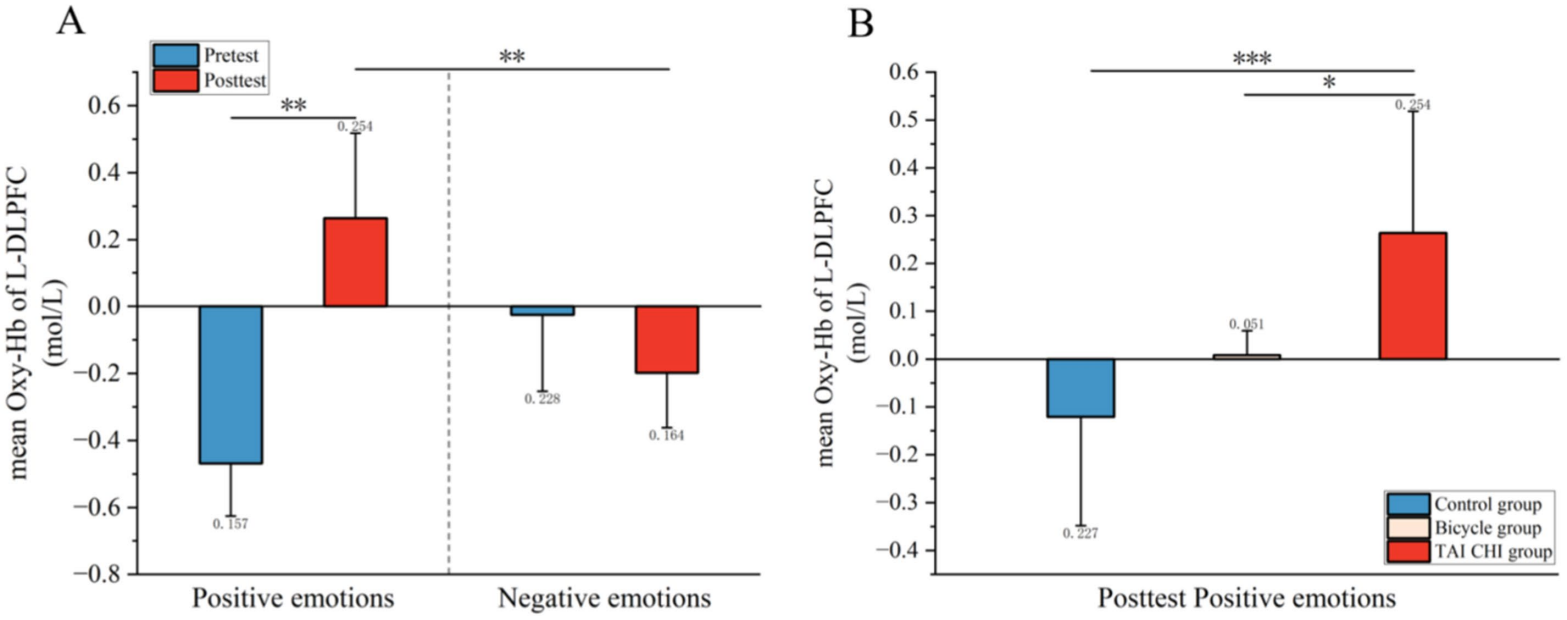
Blood oxygen activation in the L-DLPFC during emotional memory tasks. **(A)** Pre- and post-test blood oxygen activation in the L-DLPFC for the Tai Chi group in emotional memory tasks; **(B)** post-test blood oxygen activation in the L-DLPFC for the control group, cycling group, and Tai Chi group under emotional conditions.

**Figure 5:**
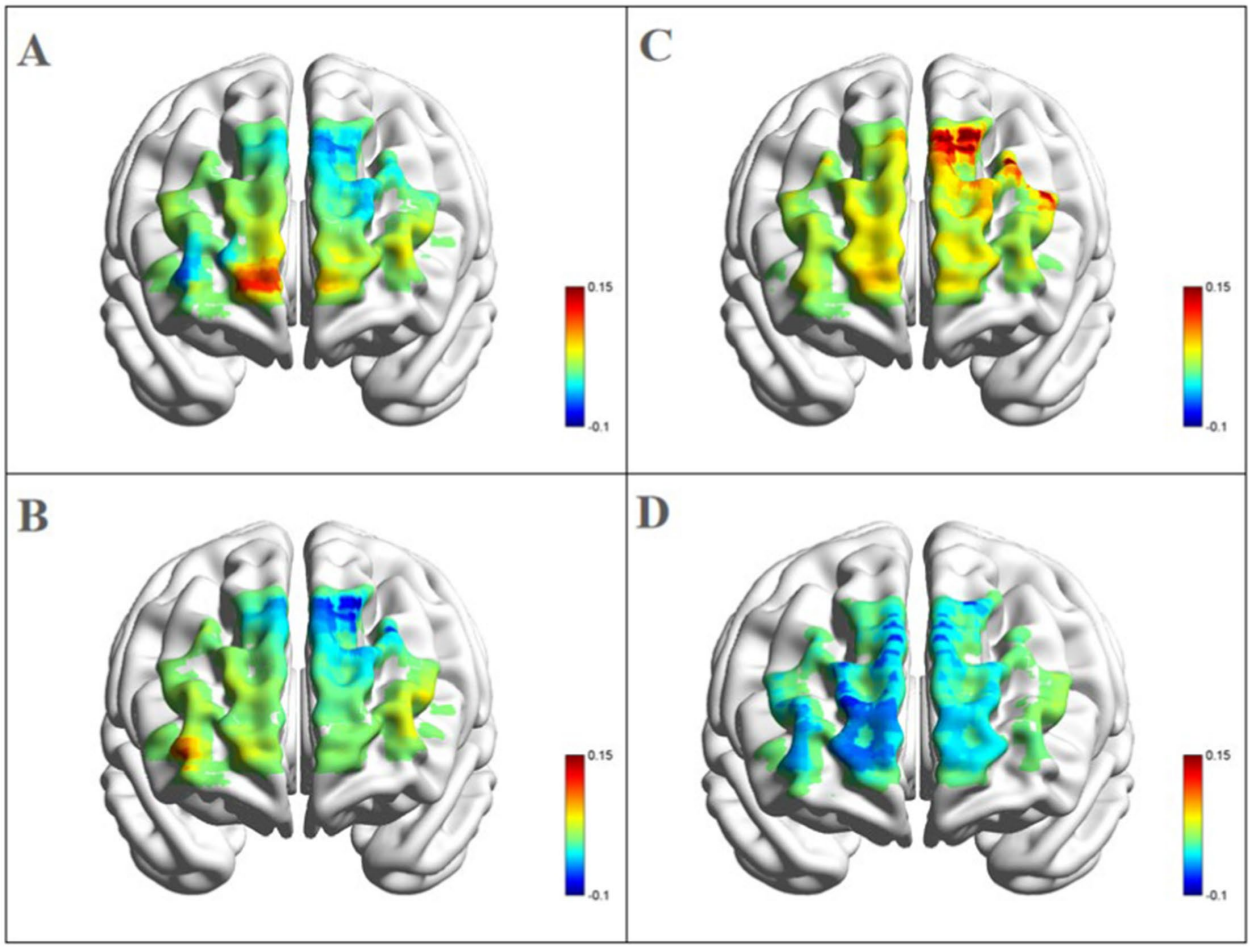
Blood oxygen activation in the PFC for the Tai Chi group during emotional memory task. **(A)** Channel activation level for the Tai Chi group during pre-test for positive emotional memory; **(B)** channel activation level for the Tai Chi group during pre-test for negative emotional memory; **(C)** channel activation level for the Tai Chi group during post-test for positive emotional memory; **(D)** channel activation level for the Tai Chi group during post-test for negative emotional memory.

**Figure 6:**
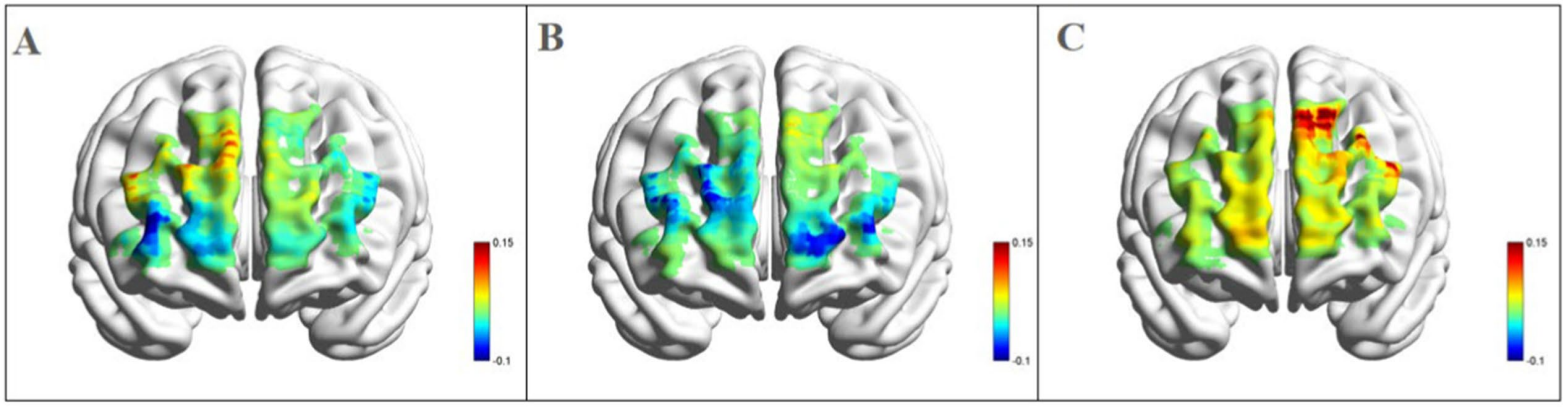
Blood oxygen activation in the PFC for different groups after intervention during positive emotional memory tasks. **(A)** Channel activation level for the control group during post-test for positive emotional memory; **(B)** channel activation level for the cycling group during post-test for positive emotional memory; **(C)** channel activation level for the Tai Chi group during post-test for positive emotional memory.

*Left dorsolateral prefrontal cortex:* as shown in Figures 4, a significant three-way interaction effect was observed among time, group, and emotional memory on oxygenation levels in L-DLPFC [*F*(2, 33) = 3.779, *p* < 0.05, *η*^2^ = 0.002]. Further analysis of simple effects revealed that under the condition of positive emotional memory, there was a significant main effect of the Tai Chi group [*F*(1, 33) = 7.957, *p* < 0.01, *η*^2^ = 0.194]. Post-hoc analysis indicated that after short-term Tai Chi intervention, the Tai Chi group exhibited a noteworthy increase in L-DLPFC Oxy-Hb concentration (0.264 ± 0.057, *p* < 0.01) compared to pre-intervention levels (−0.470 ± 0.098). Following the intervention, there was still a significant main effect of the Tai Chi group [*F*(1, 33) = 8.650, *p* < 0.01, *η*^2^ = 0.208]. Post-hoc analysis demonstrated that after the intervention, the Tai Chi group exhibited a significantly higher Oxy-Hb concentration in the L-DLPFC under positive emotional memory (0.264 ± 0.057) compared to negative emotional memory (−0.020 ± 0.085, *p* < 0.01; Figure 5). Additionally, a significant main effect was observed among different groups post-intervention [*F*(2, 33) = 11.695, *p* < 0.001, *η*^2^ = 0.415]. Further *Post-hoc* analysis revealed that under the condition of positive emotional memory, the Tai Chi group displayed significantly higher L-DLPFC Oxy-Hb concentration (0.264 ± 0.057) than both the cycling group (0.008 ± 0.057, *p* < 0.05) and the control group (−0.122 ± 0.057, *p* < 0.001). No significant difference was found between the cycling group and the control group (*p* > 0.05; Figure 6).

The main effect of the group was not significant [*F*(2, 33) = 0.926, *p* > 0.05, *η*^2^ = 0.053]. Similarly, the main effect of time was also not significant [*F*(1, 33) = 0.782, *p* > 0.05, *η*^2^ = 0.023], and there was no significant interaction effect between time and group [*F*(2, 33) = 1.353, *p* > 0.05, *η*^2^ = 0.076]. Additionally, the main effect of emotional memory type did not show statistical significance [*F*(1, 33) = 2.437, *p* > 0.05, *η*^2^ = 0.069], nor did the interaction effect between emotional memory type and group [*F*(2, 33) = 0.549, *p* > 0.05, *η*^2^ = 0.032], or the interaction effect between time and emotional memory type [*F*(1, 33) = 0.058, *p* > 0.05, *η*^2^ = 0.186].

*Right dorsolateral prefrontal cortex:* the main effect of the group did not reach statistical significance [*F*(2, 33) = 1.947, *p* > 0.05, *η*^2^ = 0.107]. Similarly, the main effect of time was not significant either [*F*(1, 33) = 0.677, *p* > 0.05, *η*^2^ = 0.020], and there was no significant interaction effect between time and group [*F*(2, 33) = 1.682, *p* > 0.05, *η*^2^ = 0.093]. Furthermore, the main effect of emotional memory type showed no statistical significance either [*F*(1, 33) = 1.728, *p* > 0.05, *η*^2^ = 0.050], nor did the interaction effect between emotional memory type and group [*F*(2, 33) = 1.650, *p* > 0.05, *η*^2^ = 0.048], or the interaction effect between time and emotional memory type [*F*(1, 33) = 1.650, *p* > 0.05, *η*^2^ = 0.048]. Finally, the three-way interaction effect among time, group, and emotional memory type was also non-significant [*F*(2, 33) = 0.351, *p* > 0.05, *η*^2^ = 0.021].

*Left frontal pole region:* the main effect of the group was not significant [*F*(2, 33) = 1.266, *p* > 0.05, *η*^2^ = 0.071], and the main effect of time was also not statistically significant [*F*(1, 33) = 0.058, *p* > 0.05, *η*^2^ = 0.002]. Additionally, there was no significant interaction effect between time and group [*F*(2, 33) = 0.051, *p* > 0.05, *η*^2^ = 0.003]. The main effect of emotional memory type was also not significant [*F*(1, 33) = 1.614, *p* > 0.05, *η*^2^ = 0.047], as well as the interaction effect between emotional memory type and group [*F*(2, 33) = 0.217, *p* > 0.05, *η*^2^ = 0.013]. Moreover, the interaction effect between time and emotional memory type was also not significant [*F*(1, 33) = 0.022, *p* > 0.05, *η*^2^ = 0.001]. Lastly, the three-way interaction effect among time, group, and emotional memory type did not yield a significant result either [*F*(2, 33) = 0.533, *p* > 0.05, *η*^2^ = 0.031].

*Right frontal pole region:* the main effect of the group was not significant [*F*(2, 33) = 2.108, *p* > 0.05, *η*^2^ = 0.113], and the main effect of time was also not significant [*F*(1, 33) = 1.063, *p* > 0.05, *η*^2^ = 0.031]. Additionally, there was no significant interaction effect between time and group [*F*(2, 33) = 0.014, *p* > 0.05, *η*^2^ = 0.091]. The main effect of emotional memory type also failed to reach significance [*F*(1, 33) = 0.293, *p* > 0.05, *η*^2^ = 0.063], as well as the interaction effect between emotional memory type and group [*F*(2, 33) = 3.574, *p* > 0.05, *η*^2^ = 0.098]. Furthermore, the interaction effect between time and emotional memory type was not significant [*F*(1, 33) = 3.574, *p* > 0.05, *η*^2^ = 0.098]. Similarly, the three-way interaction effect among time, group, and emotional memory type did not reach statistical significance either [*F*(2, 33) = 0.199, *p* > 0.05, *η*^2^ = 0.093].

### 3.3 Correlation between behavioral and neurobiological data

**Figure 7:**
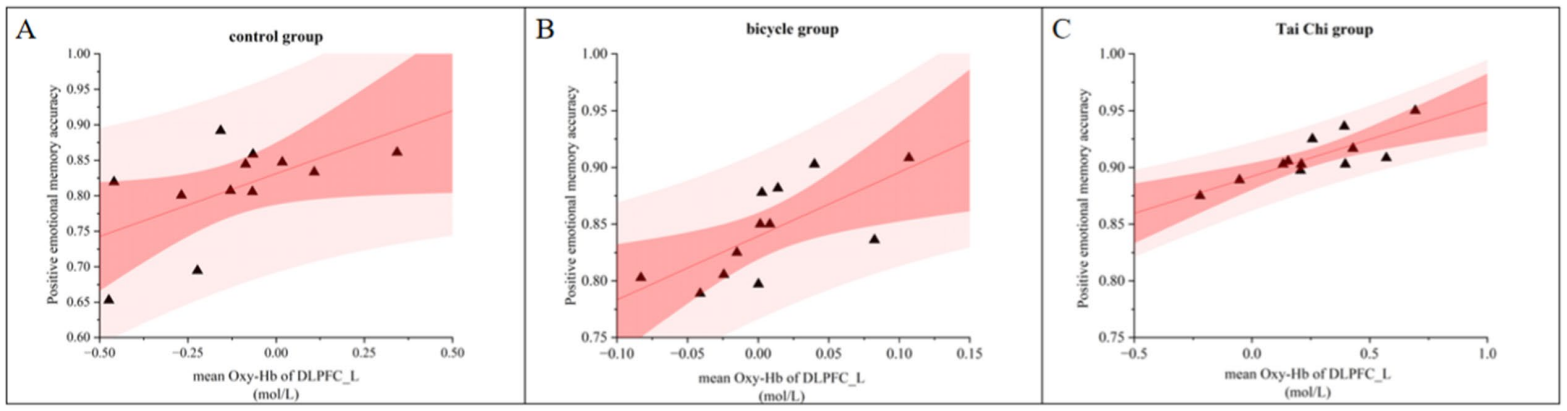
Correlation between accuracy and L-DLPFC Oxy-Hb activation during positive emotional memory post-intervention across different groups. **(A)** Control group. **(B)** Bicycle group. **(C)** Tai Chi group.

A correlation analysis was conducted to examine the relationship between the accuracy of positive emotional memory and the Oxy-Hb concentration in the L-DLPFC during the tasks involving emotional memory (Figure 7). The post-intervention correlation analysis between behavioral data and hemodynamic responses in the L-DLPFC across different groups revealed a significant positive correlation between mean Oxy-Hb concentration and emotional memory accuracy (r = 0.723, *p* < 0.001, *N* = 36). Higher levels of activation in the L-DLPFC were associated with better behavioral performance.

In the control group, a significant positive correlation was found between the Oxy-Hb concentration in the L-DLPFC and emotional memory accuracy after the intervention (r = 0.579, *p* < 0.05, *N* = 12; Figure 7A). Similarly, in the cycling group, a significant positive correlation was observed post-intervention (r = 0.690, *p* < 0.05, *N* = 12; Figure 7B). In the Tai Chi group, there was a significant positive correlation between the Oxy-Hb concentration in the L-DLPFC and emotional memory accuracy after intervention (r = 0.813, *p* < 0.01, *N* = 12; Figure 7C).

## 4 Discussion

This study utilized fNIRS technology and a mixed experimental design to investigate the modulatory effects of acute moderate-intensity Tai Chi and power cycling on emotional memory and PFC hemodynamics. The findings revealed that: (1) In the control and cycling groups, the accuracy of negative emotional memory was significantly higher than that of positive emotional memory, while no significant difference was observed in the Tai Chi group. Additionally, the accuracy of positive emotional memory in the Tai Chi group was significantly higher than in the control group. (2) For positive emotional memory, both the cycling and Tai Chi groups showed significantly improved post-intervention accuracy compared to pre-intervention, whereas no significant difference was found in the control group. (3) In the Tai Chi group, post-intervention Oxy-Hb concentration in the L-DLPFC significantly increased for positive emotional memory compared to pre-intervention. This concentration was also significantly higher for positive emotional memory than for negative emotional memory, and higher than that in the cycling and control groups. (4) A significant positive correlation was found between increases in L-DLPFC Oxy-Hb concentration and accuracy in emotional memory tasks, with a stronger correlation in the Tai Chi group compared to the cycling and control groups. These findings support the association between acute Tai Chi exercise, emotional memory performance, and Oxy-Hb concentration in the PFC, providing empirical evidence for the relationship between different forms of acute exercise and emotional memory among college students.

### 4.1 Effects of different exercises on behavioral performance in emotional memory tasks

The interaction effect between time and group indicates that both the Tai Chi and cycling groups exhibited significantly higher accuracy in emotional memory after the intervention compared to the control group. Additionally, both groups showed significant improvements in accuracy from pre- to post-intervention. Although there was no statistically significant difference in accuracy between the Tai Chi and cycling groups after the intervention, Tai Chi had a more pronounced impact on enhancing emotional memory accuracy compared to cycling. Previous research has indicated that among older adults, engaging in 6 min of cycling at 70% VO_2_max during the memory consolidation period resulted in enhanced emotional memory (assessed through IAPS stimuli) as opposed to resting in a seated position (Huellemann et al., 2021). Similarly, among young adults, a single bout of intense walking exercise (60–85% VO_2_max) during the memory consolidation period enhanced emotional memory compared to slow walking (Keyan and Bryant, 2017b). Both studies implemented exercise after the presentation of memory stimuli, specifically during the early stage of memory consolidation. In contrast to these studies, our research introduced a bout of exercise prior to memory encoding. This timing was chosen based on previous experimental studies on non-emotional episodic memory which have suggested that acute exercise before memory encoding may be more advantageous than other time periods (Frith et al., 2017; Sng et al., 2018). Consistent with our findings, acute aerobic exercise has been demonstrated to enhance emotional memory function. From a cognitive psychology perspective, exercise acts as a stressor that elicits heightened arousal levels and cognitive engagement as its intensity increases. Moderate-intensity exercise is thought to enhance activity in the sympathetic-adrenal system and the hypothalamic–pituitary–adrenal axis, potentially leading to elevated concentrations of norepinephrine, dopamine, adrenocorticotropic hormone (ACTH), and cortisol in the brain. Consequently, this optimization may improve performance in memory tasks (McMorris et al., 2011).

The interaction effect between emotional memory type and group shows that in the control and cycling groups, the accuracy of negative emotional memory is significantly higher than that of positive emotional memory. Previous research suggests that emotionally intense memories are more likely to be retained for extended periods compared to neutral memories due to their typically associated stronger physiological and psychological responses (Hamann, 2001; LaBar and Cabeza, 2006), which aligns with the findings of this study. In the Tai Chi group, there is no significant difference between the accuracy of positive and negative emotional memories, but the accuracy of positive emotional memory is significantly higher than that of the control group. Tai Chi incorporates elements of mindfulness known for effectively enhancing positive emotions (S. Zhang et al., 2019). Attention plays a crucial role as a tool and key component in emotion regulation (Wadlinger and Isaacowitz, 2011). The integration of attention, movement, posture control, and visual imagery in Tai Chi may offer additional cognitive stimulation. Tai Chi necessitates practitioners to concentrate on specific postures and sequences of movements, thereby enhancing visual attention (visual span) (Lam et al., 2011). For instance, during Tai Chi practice, practitioners are required to achieve relaxation, calmness, and focused attention on breathing and sensory experiences, which serves as a form of attention training. Attention deployment is a fundamental mechanism for regulating emotions and plays a significant role in the preceding emotion regulation strategies that influence the generation, maintenance, or modification of emotions (Boelens et al., 2021). In comparison to traditional exercises, Tai Chi involves more comprehensive attention control training. The broaden-and-build theory of positive emotions posits that attention can be redirected or expanded. Positive emotions broaden the capacity for visual attention, allowing individuals to process more information (Rowe et al., 2007). Therefore, positive emotions are associated with an increased breadth of attention, whereas negative emotions are linked to a narrowed focus. Tai Chi training significantly enhances cognitive functions related to the storage, encoding, and retrieval of positive emotional memories. The absence of significant changes in reaction time may be attributed to the fact that reaction time is primarily influenced by neural conduction speed and muscle response speed, which do not undergo substantial alterations following a single short-term exercise session. Additionally, acute exercise might induce a certain degree of fatigue that could counterbalance the potential beneficial effects on reaction speed.

### 4.2 Effects of different acute exercises on Oxy-Hb concentration in ROI

This study used fNIRS to measure the impact of different exercise modalities on the concentration of Oxy-Hb in the L-DLPFC. It was found that both acute Tai Chi and moderate-intensity power cycling significantly elevated Oxy-Hb concentration in this region, especially during tasks involving positive emotional memory, with Tai Chi demonstrating a more pronounced effect. Given the crucial role of the PFC in encoding and retrieving emotional events (LaBar and Cabeza, 2006), repetitive transcranial magnetic stimulation (rTMS) of the L-DLPFC has been shown to enhance the retrieval of positive emotional information (Balconi and Ferrari, 2012). Considering that the L-DLPFC plays a significant role in positive emotional memory and cognition (Kohn et al., 2014), these results provide novel evidence from cognitive neuroscience to support the findings of this study, reinforcing the strong correlation between positive emotional memory and blood oxygen activation in the L-DLPFC. However, the psychological mechanisms underlying these effects require further analysis. Specifically, Tai Chi’s impact on positive emotional memory may be attributed to enhanced attentional control, emotional regulation, and psychological relaxation. In addition, studies have shown that Tai Chi practitioners exhibit higher hippocampal density and increased regional homogeneity (ReHo) in the hippocampus and parahippocampal regions compared to walking groups. This enhanced ReHo is positively correlated with improved memory performance (Ji et al., 2017; Yue et al., 2020). This aligns with evidence that Tai Chi practice induces structural and functional changes in the PC (Wei et al., 2014) and improves memory (Tao et al., 2016).

Tai Chi aligns with theories indicating that moderate-intensity acute exercise can enhance cognitive function by activating neural substrates, particularly through increased cortical activation in the L-DLPFC (Byun et al., 2014; Yanagisawa et al., 2010). The practice of Tai Chi integrates mental focus and breathing control with physical movements to achieve a harmonious equilibrium between mind and body (Lan et al., 2013). This integration enhances attentional control and fosters emotional regulation, reducing stress and anxiety levels while promoting positive emotions (Zhou et al., 2018). Such benefits may underlie Tai Chi’s unique ability to improve the accuracy of positive emotional memory. During the positive emotional memory task, participants practicing Tai Chi showed significantly increased Oxy-Hb concentration in the L-DLPFC. This increase is likely linked to Tai Chi’s meditative and relaxation components, which enhance capabilities for emotional regulation and facilitate effective processing of positive emotional information during memory tasks (Yao et al., 2021). In contrast, power cycling lacks these mindfulness-based elements, which may account for its comparatively lower effect on increasing Oxy-Hb concentration in the L-DLPFC. Furthermore, tasks requiring attention shifting have been demonstrated to activate the parietal and L-DLPFC cortices (McMorris et al., 2011). Tai Chi combines physical movement with meditative focus, which not only improves attentional control but also enhances the synchronization between different brain regions involved in emotional memory tasks (Tang et al., 2017). This integrative nature may help establish a solid neural foundation for emotional memory accuracy. Emerging evidence suggests that Tai Chi alleviates symptoms of depression, anxiety, and psychological stress while significantly enhancing positive emotions (Wu et al., 2022; Zou et al., 2018). The breathing techniques and structured movements of Tai Chi promote emotional regulation by activating the L-DLPFC, further supporting its distinct role in fostering positive emotional memory.

In summary, acute Tai Chi improves the accuracy of positive emotional memory by increasing Oxy-Hb concentration in the L-DLPFC. This effect can be attributed to the unique integration of mind and body, which enhances cortical hemodynamics, attentional control, and emotional regulation. Compared to power cycling, Tai Chi provides a more comprehensive approach to promoting emotional memory, making it a valuable tool for future research and practical applications in this area.

### 4.3 Limitations

Tai Chi appears to have a greater impact on cortical hemodynamics compared to general aerobic exercises. Future research involving diverse populations and larger sample sizes is needed to confirm these findings (Wang et al., 2023). Long-term intervention studies are also essential to assess whether the effects of Tai Chi on emotional memory are sustained over time or transient. A long-term follow-up would help determine the sustainability of the effects of Tai Chi on emotional memory and assess whether the observed benefits are enduring or transient. Furthermore, it is important to explore whether the effects of Tai Chi are unique to this practice or if they could be generalized to other forms of moderate-intensity exercise. Future studies could also benefit from dual-modal imaging techniques, such as combining fNIRS with electroencephalography (EEG) (Wang et al., 2023). This approach would enhance the sensitivity in detecting brain activity changes, providing more accurate assessments of emotional memory. Additionally, the study’s limitations extend beyond sample size and include the lack of consideration for sex differences and cultural backgrounds, both of which may influence the effects of Tai Chi interventions. For instance, sex-specific factors, such as hormonal fluctuations and phases of the menstrual cycle, are known to affect emotional memory and exercise responses (Nakamura et al., 2023; Sacher et al., 2013). Similarly, cultural attitudes toward exercise and Tai Chi practice may modulate intervention outcomes. Future research should investigate these aspects to provide a more comprehensive understanding of the mechanisms involved. Cross-cultural studies and subgroup analyses based on sex could offer valuable insights into how these variables interact with Tai Chi interventions. Including these variables will offer a more nuanced understanding of how Tai Chi affects emotional memory mechanisms.

## 5 Conclusion

This study highlights the effectiveness of acute exercise, specifically Tai Chi, in activating brain regions associated with positive emotions and memory, as reflected by increased Oxy-Hb concentration in the L-DLPFC. These findings provide evidence for the neural mechanisms underlying cognitive and emotional benefits of exercise and suggest potential applications in interventions and cognitive training. While the participants in this study were healthy young adults, it is essential to consider how these findings might translate to broader populations. Future research should explore the applicability of Tai Chi to older adults or individuals with emotional or cognitive disorders, such as anxiety or depression, to determine its broader relevance. Older adults, in particular, may benefit from the impact and mindfulness-based nature of Tai Chi, which could improve both physical and emotional health. Additionally, investigating how Tai Chi can be incorporated into therapeutic programs for emotional disorders could provide valuable insights into its broader clinical utility. Furthermore, potential cultural or demographic influences on the outcomes of this study warrant attention. As Tai Chi is deeply rooted in Chinese culture, it is crucial to evaluate its acceptability and effectiveness in populations with diverse cultural backgrounds. Exploring cultural adaptations or alternative forms of mind–body exercises may enhance the generalizability and applicability of the findings. Future studies should also consider factors such as socioeconomic status, education level, and access to exercise programs, which may influence the feasibility and efficacy of implementing Tai Chi in different settings. Based on these results, it is recommended that practitioners consider incorporating Tai Chi into exercise interventions aimed at improving cognitive and emotional outcomes, particularly in diverse and at-risk populations.

## Data Availability

The datasets presented in this study can be found in online repositories. The names of the repository/repositories and accession number(s) can be found in the article/Supplementary material.
